# Risk factors and secondary care utilisation in a primary care population with non-tuberculous mycobacterial disease in the UK

**DOI:** 10.1007/s10096-018-3402-8

**Published:** 2018-10-27

**Authors:** Eleanor L. Axson, Navdeep Bual, Chloe I. Bloom, Jennifer K. Quint

**Affiliations:** Respiratory Epidemiology, Occupational Medicine and Public Health, National Heart and Lung Institute, Imperial College London, London, SW3 6LR UK

**Keywords:** Epidemiology, Risk factors, NTM, COPD, Bronchiectasis

## Abstract

Prior research has identified risk factors associated with developing non-tuberculous mycobacterial disease (NTMD); we identified risk factors and secondary care utilisation of NTMD patients in the UK. This was a matched case-control study using electronic healthcare records from Clinical Practice Research Datalink from 2006 to 2016. NTMD was defined using prescription data and Read codes, based on international guidelines. Risk factors for NTMD were investigated using conditional logistic regression within a representative general population. All-cause secondary care utilisation (combined inpatient, outpatient, emergency visits) was investigated for participants with linked Hospital Episode Statistics (HES), using incidence rate ratio (IRR) from 2007 to 2015. We identified 1225 individuals with NTMD. A subset of individuals (426 patients) were eligible for linkage with HES. In the adjusted model, risk factors most strongly associated with an increased likelihood of NTMD included previous tuberculosis (OR 69.0; 47.7–99.8); bronchiectasis (OR 23.3; 12.4–43.9); lung cancer (OR 14.9; 3.98–55.7); oral corticosteroids (OCS; OR 7.28; 4.94–10.7); immunosuppressive (excluding corticosteroids) medication (OR 3.05; 1.15–8.10); being underweight (odds ratio (OR) 2.92; 95% CI 1.95, 4.36); and rheumatoid arthritis (OR 2.12; 1.05–4.27). NTMD patients had significantly higher rates of all-cause secondary care utilisation than non-NTMD patients (IRR 5.80; 5.14–6.46). Using a representative adult population, we identified prior TB, bronchiectasis, lung cancer, immunosuppressive medication, and OCS as the risk factors associated with the highest odds of developing NTMD in the UK. Patients with NTMD experienced nearly six times more all-cause secondary care events following their NTMD diagnosis than patients without NTMD.

## Background

Non-tuberculous mycobacterium (NTM) are naturally occurring bacteria ubiquitous in the environment, found in water and soil [1]. There is great geographic variation in NTM species [2], and, while NTM do not normally cause disease, high-risk populations have been identified [3, 4]. Disease from NTM (NTMD) can manifest throughout the body, but pulmonary infections are the most common [5].

Established risk factors for NTMD include low body fat [6], bronchiectasis [7], and history of tuberculosis (TB) [3]. While immunosuppression is accepted as a risk factor [8], studies have yet to tease out the role of diseases—such as rheumatoid arthritis (RA) [9], chronic obstructive pulmonary disease (COPD) [3, 10], and asthma [11]—as opposed to their treatments—such as inhaled corticosteroids (ICS) and oral corticosteroids (OCS) [3, 10]. Additionally, some studies have found higher proportions of NTMD patients being current or former smokers, having gastroesophageal reflux disease (GORD), chronic kidney disease (CKD), and lung cancer [11].

Previous studies have also highlighted that patients with NTMD utilise greater secondary care resources than those without NTMD, with Germany seeing a 5.9% increase per year in NTMD-related hospitalisations from 2009 to 2014 [12] and a US study finding NTMD patients at a greater risk of hospitalisation [13]. Healthcare expenditure for NTMD patients is significantly higher than for those without NTMD, placing noteworthy burden on healthcare systems [12, 13].

This is the first study to examine risk factors and secondary care utilisation for NTMD patients in a representative UK adult population using primary care records. Additionally, we examine both risk factors and secondary care utilisation in a subpopulation of patients with underlying chronic respiratory diseases (CRDs) including asthma, bronchiectasis, COPD, cystic fibrosis (CF), and interstitial lung disease (ILD).

## Methods

### Data source

The Clinical Practice Research Datalink (CPRD) provides access to detailed, anonymised primary care records for 6.8% of the UK population, shown to be representative of the UK population in terms of sex, BMI, and ethnicity [14]. Linkage of CPRD data with Hospital Episode Statistics (HES) is available for ~ 75% of practices in England [14]. HES datasets provide data from inpatient, outpatient, and accident and emergency (A&E) attendance including patient demographics, diagnoses, admission, and discharge [15].

### Defining NTMD

The identification of people with NTMD included antimycobacterial prescription data and Read codes—a form of clinical classification describing patient conditions, medications, and diagnoses—for NTMD between 01/01/2006 and 31/12/2016. The cohort comprised patients with evidence of appropriate treatment/monitoring of NTMD as determined by international guidelines for the treatment and management of NTMD [5, 16, 17]. Patients must have met the following criteria [18]:

1. Have 3+ NTM sputum samples sent ≥ 3 months apart over 2 years;

OR

2. Be taking a guideline-recommended [5, 16, 17] multi-drug regimen with 3+ consecutive prescriptions within 100 days of each other;

OR

3. Be taking ≥ 2 drugs with 3+ consecutive prescriptions within 100 days of each other identified from guidelines as being used to treat NTMD [5, 16, 17] that must include at least one of (a) isoniazid, (b) ethambutol, or (c) rifampicin/rifabutin in combination with isoniazid or ethambutol.

Due to the nature of coding in CPRD, it was not possible to identify infection location and thus we refer to NTMD not NTM lung disease though we suspect the majority of patients have NTM in the lungs.

### Defining the study populations

**Fig. 1:**
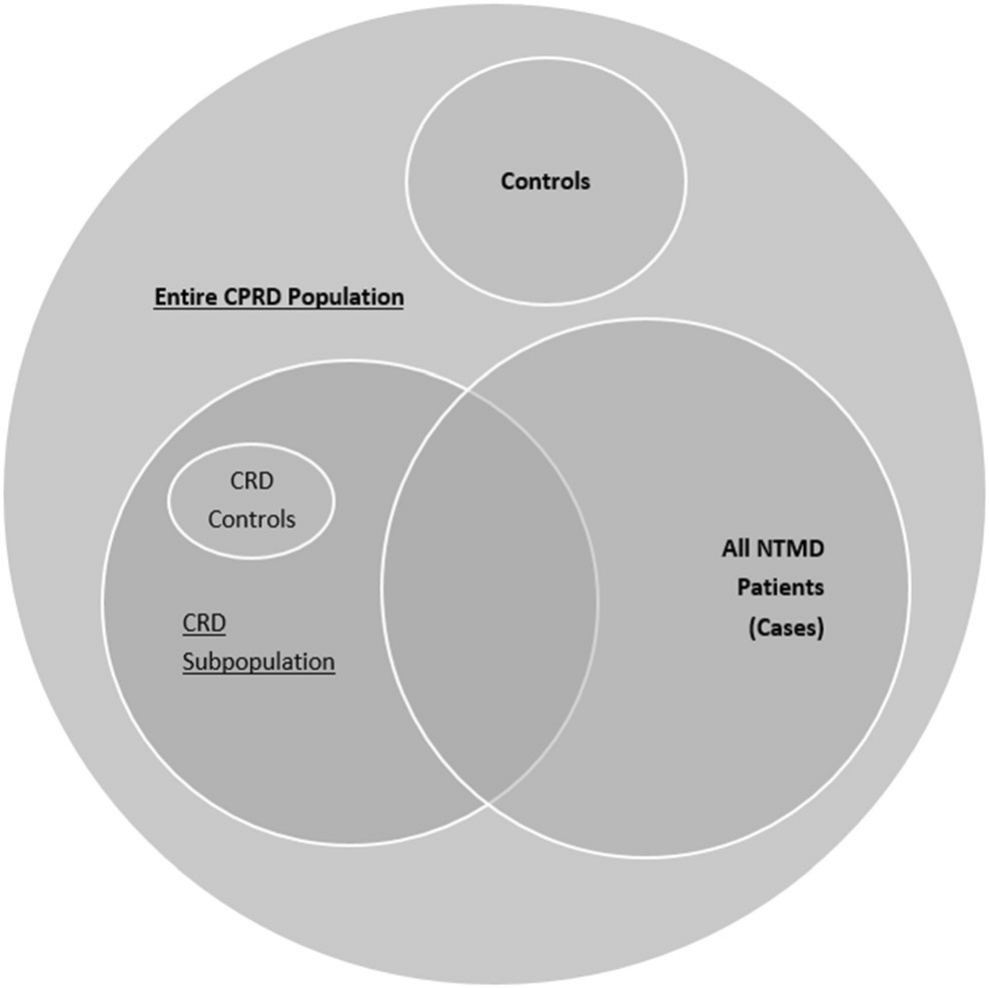
Defining the study populations. All cases and controls were adults aged 18+, whose practice data was deemed to be ‘up-to-standard’ for research purposes by CPRD. Start of follow-up was defined as the latest of (1) study start (01/01/2007); (2) patient’s 18th birthday; (3) patient’s current record date; (4) practice’s ‘up-to-standard’ date; or (5) the index date of the case. End of follow-up was defined as the first of (1) study end (31/12/2015); (2) patient’s last record collection date; (3) patient transferring out of practice; or (4) patient death

Our population consisted of all identified NTMD cases matched 1:6 on sex, age (5 years), and general practice to randomly selected controls from the CPRD population (Fig. 1). We also investigated a subpopulation (CRD subpopulation) of NTMD cases with underlying CRD also matched 1:6 on sex, age (5 years), and general practice to randomly selected controls from the CPRD population with CRD (Fig. 1). CRD included asthma, bronchiectasis, CF, COPD, and ILD. Asthma [19] and COPD [20] patients were identified using validated algorithms. The Read codes used to identify bronchiectasis [21], ILD [22], and CF [18] patients have been published. In both populations, cases and controls were matched on the index date, the first date of NTMD, for the case.

### Demographics

We included age (years), sex (male/female), and smoking status (never/not recorded, former, and current). Body mass index (BMI; kg/m^2^) was divided into underweight (< 18.5), healthy weight (18.5–24.9), overweight (25.0–29.9), and obese (> = 30).

### Analysis of risk factors

Risk factors were determined using previous literature on NTMD (Table 1; excluding sex and age) [3, 4, 6, 7, 9–11]. Patients could have more than one risk factor and only conditions coded any time prior to the index date were considered risk factors. Current immunosuppressive, ICS, and OCS use were defined as having had ≥ 2 prescriptions within 6 months of the index date. For chronic conditions, patients were considered to have the condition from the time of the first Read code.

**Table 1:** Descriptive statistics for the general population and chronic respiratory disease (CRD) population for cases and controls

	NTMD cases (*n* = 1225)	Controls (*n* = 7308)	NTMD cases in CRD subpopulation (*n* = 463)	Controls in CRD subpopulation (*n* = 2764)
Female	584 (47.7)	3504 (47.7)	218 (47.1)	1308 (47.1)
Mean age ± SD (years)	55.2 ± 18.5	55.2 ± 18.6	63.6 ± 14.5	63.6 ± 14.4
Males	55.5 ± 17.5	55.5 ± 17.5	62.9 ± 13.9	62.9 ± 13.8
Females	54.9 ± 19.7	54.9 ± 19.6	64.4 ± 15.1	64.4 ± 15.1
Smoking status				
Never smoker/not recorded	571 (46.6)	4073 (55.4)	136 (29.4)	1163 (41.9)
Current smoker	327 (36.7)	1827 (24.9)	145 (31.2)	691 (24.9)
Former smoker	327 (36.7)	1450 (19.7)	182 (39.3)	924 (33.3)
Body mass index				
Underweight (< 18.5)	124 (11.1)	151 (2.36)	64 (14.3)	49 (1.83)
Healthy weight (18.5–24.9)	533 (47.6)	2351 (36.7)	232 (51.8)	793 (29.6)
Overweight (25.0–29.9)	312 (27.9)	2351 (36.7)	106 (23.7)	962 (36.0)
Obese (> = 30)	151 (13.5)	1550 (24.2)	46 (10.3)	871 (32.6)
CRD risk factors				
Asthma	167 (13.6)	387 (5.27)	138 (29.8)	1321 (47.6)
Bronchiectasis	104 (8.49)	25 (0.34)	103 (22.3)	165 (5.94)
COPD	232 (18.9)	274 (3.73)	229 (49.5)	1029 (37.0)
Cystic fibrosis	10 (0.82)	< 5	9 (1.94)	6 (0.22)
Interstitial lung disease	30 (2.45)	29 (0.39)	20 (4.32)	76 (2.74)
Other disease risk factors				
Chronic kidney disease	131 (10.7)	423 (5.76)	33 (7.13)	276 (9.94)
Cancer, lung	17 (1.39)	7 (0.10)	12 (2.59)	15 (0.54)
Cancer, other than lung	117 (9.55)	598 (8.14)	59 (12.7)	332 (12.0)
Diabetes	144 (11.8)	663 (9.02)	51 (11.0)	374 (13.5)
Gastroesophageal reflux disease	156 (12.7)	730 (9.93)	74 (16.0)	449 (16.2)
History of tuberculosis	518 (42.3)	76 (1.03)	171 (36.9)	54 (1.94)
Rheumatoid arthritis	40 (3.27)	67 (0.91)	22 (4.75)	55 (1.98)
Medication risk factors				
Immunosuppressive use*	16 (1.31)	35 (0.48)	8 (1.73)	11 (0.40)
Inhaled corticosteroids	251 (20.5)	409 (5.56)	228 (49.2)	1137 (40.9)
Oral corticosteroids	180 (14.7)	127 (1.73)	125 (27.0)	155 (5.58)

Non-tuberculous mycobacterial disease (NTMD). CRD including asthma, bronchiectasis, cystic fibrosis, chronic obstructive pulmonary disease (COPD), and interstitial lung disease

*SD s*tandard deviation

Data are presented as number of patients (%) unless otherwise specified

*Excluding corticosteroids

Conditional logistic regression was used to estimate the association between risk factors and incident NTMD. A disease-only model included all disease risk factors for NTMD, but no medications. A treatment-adjusted model included all risk factors, both diseases and medications, in order to identify whether diseases or treatment were driving NTMD risk.

### Analysis of all-cause secondary care utilisation

Using a subset of patients with linked HES data available, all-cause secondary care utilisation was defined as the cumulative number of inpatient stays, outpatient visits, and A&E attendance from 2007 to 2015 in England. These matched cohort analyses used the populations identified above (Fig. 1). The average incidence rate ratio (IRR) comparing all-cause secondary care utilisation between patients with NTMD to patients without NTMD from 2007 to 2015 was analysed. Similar analysis of mortality was planned, but was unable to be completed due to small numbers of deaths.

All statistical analyses were performed in STATA version 15 (StataCorp LP, College Station, TX, USA).

## Results

### Description of study populations

There were 1225 cases of NTMD identified in the general population and 463 cases identified in the CRD subpopulation from 2006 to 2016 (Table 1). A subset of individuals (426 general; 163 CRD) were eligible for linkage with HES.

### Risk factors for NTMD in the general population

Results of the crude, disease-only, and treatment-adjusted regression models can be seen in Table 2. Cancers other than lung were not significantly associated with NTMD in any of the models. All other risk factors were significantly associated with increased NTMD in all the models (Table 2).

**Table 2:** Conditional logistic regression models for non-tuberculous mycobacterial disease (NTMD) risk factors in the whole population

	Crude OR (95% CI)	*p* value	Model w/o treatments OR (95% CI)	*p* value	Model with treatments OR (95% CI)	*p* value
Smoking status						
Never smoker/not recorded	Reference		Reference		Reference	
Current smoker	1.30 (1.12, 1.51)	0.001	0.92 (0.73, 1.17)	NS	0.90 (0.71, 1.14)	NS
Former smoker	1.68 (1.44, 1.96)	< 0.0001	1.30 (1.03, 1.64)	0.029	1.23 (0.97, 1.57)	NS
Body mass index						
Underweight (< 18.5)	3.87 (2.94, 5.10)	< 0.0001	2.68 (1.81, 3.97)	< 0.0001	2.92 (1.95, 4.36)	< 0.0001
Healthy weight (18.5–24.9)	Reference		Reference		Reference	
Overweight (25.0–29.9)	0.56 (0.48, 0.66)	< 0.0001	0.62 (0.50, 0.77)	< 0.0001	0.61 (0.49, 0.76)	< 0.0001
Obese (> = 30)	0.41 (0.34, 0.52)	< 0.0001	0.40 (0.30, 0.52)	< 0.0001	0.40 (0.31, 0.53)	< 0.0001
CRD risk factors						
Asthma	2.91 (2.39, 3.54)	< 0.0001	1.80 (1.31, 2.48)	< 0.0001	1.30 (0.92, 1.85)	NS
Bronchiectasis	29.1 (18.2, 46.5)	< 0.0001	25.2 (13.7, 46.3)	< 0.0001	23.3 (12.4, 43.9)	< 0.0001
COPD	7.22 (5.86, 8.89)	< 0.0001	4.20 (3.04, 5.82)	< 0.0001	2.47 (1.69, 3.62)	< 0.0001
Interstitial lung disease	6.79 (3.98, 11.6)	< 0.0001	3.07 (1.30, 7.25)	0.011	1.97 (0.82, 4.76)	NS
Other disease risk factors						
Chronic kidney disease	2.21 (1.76, 2.78)	< 0.0001	3.21 (2.33, 4.42)	< 0.0001	3.07 (2.21, 4.27)	< 0.0001
Cancer, lung	14.6 (6.04, 35.1)	< 0.0001	12.8 (3.35, 49.1)	< 0.0001	14.9 (3.98, 55.7)	< 0.0001
Cancer, other than lung	1.22 (0.98, 1.52)	NS	1.07 (0.76, 1.51)	NS	1.03 (0.72, 1.48)	NS
Diabetes	1.37 (1.12, 1.66)	0.002	1.59 (1.19, 2.12)	0.002	1.54 (1.14, 2.08)	0.005
Gastroesophageal reflux disease	1.35 (1.11, 1.63)	0.002	1.12 (0.84, 1.50)	NS	1.08 (0.80, 1.47)	NS
History of tuberculosis	66.8 (49.2, 90.6)	< 0.0001	62.3 (43.5, 89.2)	< 0.0001	69.0 (47.7, 99.8)	< 0.0001
Rheumatoid arthritis	3.73 (2.50, 5.57)	< 0.0001	4.22 (2.32, 7.68)	< 0.0001	2.12 (1.05, 4.27)	0.036
Medication risk factors						
Immunosuppressive use*	2.74 (1.52, 4.96)	0.001			3.05 (1.15, 8.10)	0.026
Inhaled corticosteroids	4.50 (3.78, 5.36)	< 0.0001			1.51 (1.07, 2.14)	0.020
Oral corticosteroids	10.4 (8.09, 13.3)	< 0.0001			7.28 (4.94, 10.7)	< 0.0001

Chronic respiratory diseases (CRD) including asthma, bronchiectasis, cystic fibrosis, chronic obstructive pulmonary disease (COPD), and interstitial lung disease

*OR* odds ratio, *NS* not significant

Significant *p* < 0.05

*Excluding corticosteroids

### Risk factors for NTMD in the CRD subpopulation

Results of the crude, disease-only, and treatment-adjusted regression models in the CRD subpopulation can be seen in Table 3. Asthma, other cancers, diabetes, and GORD were not associated with NTMD in any of the models. COPD and ILD were significantly associated with NTMD in the crude and disease-only models, but lost significance in the treatment-adjusted model. All other risk factors were significantly associated with increased NTMD in all the models (Table 3).

**Table 3:** Conditional logistic regression models for non-tuberculous mycobacterial disease (NTMD) risk factors in the chronic respiratory disease (CRD) subpopulation

	Crude OR (95% CI)	*p* value	Model w/o treatments OR (95% CI)	*p* value	Model with treatments OR (95% CI)	*p* value
Smoking status						
Never smoker/not recorded	Reference		Reference		Reference	
Current smoker	1.87 (1.44, 2.43)	< 0.0001	1.28 (0.87, 1.89)	NS	1.25 (0.83, 1.88)	NS
Former smoker	1.75 (1.36, 2.23)	< 0.0001	1.59 (1.12, 2.25)	0.009	1.48 (1.02, 2.13)	0.037
Body mass index						
Underweight (< 18.5)	5.15 (3.30, 8.06)	< 0.0001	4.59 (2.57, 8.18)	< 0.0001	4.70 (2.58, 8.55)	< 0.0001
Healthy weight (18.5–24.9)	Reference		Reference		Reference	
Overweight (25.0–29.9)	0.35 (0.27, 0.43)	< 0.0001	0.43 (0.31, 0.59)	< 0.0001	0.42 (0.30, 0.59)	< 0.0001
Obese (> = 30)	0.17 (0.12, 0.23)	< 0.0001	0.22 (0.14, 0.33)	< 0.0001	0.20 (0.13, 0.30)	< 0.0001
CRD risk factors						
Asthma	0.81 (0.64, 1.03)	NS	0.98 (0.71, 1.35)	NS	0.82 (0.58, 1.16)	NS
Bronchiectasis	6.18 (4.58, 8.34)	< 0.0001	6.63 (4.42, 9.94)	< 0.0001	6.88 (4.46, 10.6)	< 0.0001
COPD	2.38 (1.91, 2.97)	< 0.0001	1.74 (1.27, 2.38)	0.001	1.29 (0.92, 1.82)	NS
Interstitial lung disease	2.18 (1.29, 3.68)	0.004	2.66 (1.24, 5.70)	0.012	2.08 (0.92, 4.73)	NS
Other disease risk factors						
Chronic kidney disease	0.66 (0.44, 0.98)	0.041	0.78 (0.45, 1.33)	NS	0.81 (0.46, 1.45)	NS
Cancer, lung	5.18 (2.35, 11.4)	< 0.0001	9.23 (2.99, 28.5)	< 0.0001	8.83 (2.59, 30.1)	< 0.0001
Cancer, other than lung	1.08 (0.80, 1.47)	NS	0.86 (0.54, 1.39)	NS	0.89 (0.53, 1.48)	NS
Diabetes	0.79 (0.58, 1.08)	NS	1.44 (0.95, 2.19)	NS	1.20 (0.76, 1.88)	NS
Gastroesophageal reflux disease	0.99 (0.75, 1.30)	NS	1.04 (0.71, 1.51)	NS	0.92 (0.62, 1.37)	NS
History of tuberculosis	35.2 (23.4, 52.8)	< 0.0001	29.9 (18.7, 48.0)	< 0.0001	36.1 (21.7, 60.2)	< 0.0001
Rheumatoid arthritis	2.50 (1.50, 4.17)	< 0.0001	5.41 (2.62, 11.2)	< 0.0001	2.49 (1.04, 5.97)	0.041
Medication risk factors						
Immunosuppressive use*	4.36 (1.76, 10.8)	0.002			9.23 (2.12, 40.1)	0.003
Inhaled corticosteroids	1.46 (1.18, 1.79)	< 0.0001			1.52 (1.11, 2.08)	0.008
Oral corticosteroids	6.60 (5.00, 8.70)	< 0.0001			6.17 (4.17, 9.13)	< 0.0001

CRD including asthma, bronchiectasis, cystic fibrosis, chronic obstructive pulmonary disease (COPD), and interstitial lung disease

*OR* odds ratio, *NS* not significant

Significant *p* < 0.05

*Excluding corticosteroids

### Secondary care utilisation in the general population

**Fig. 2:**
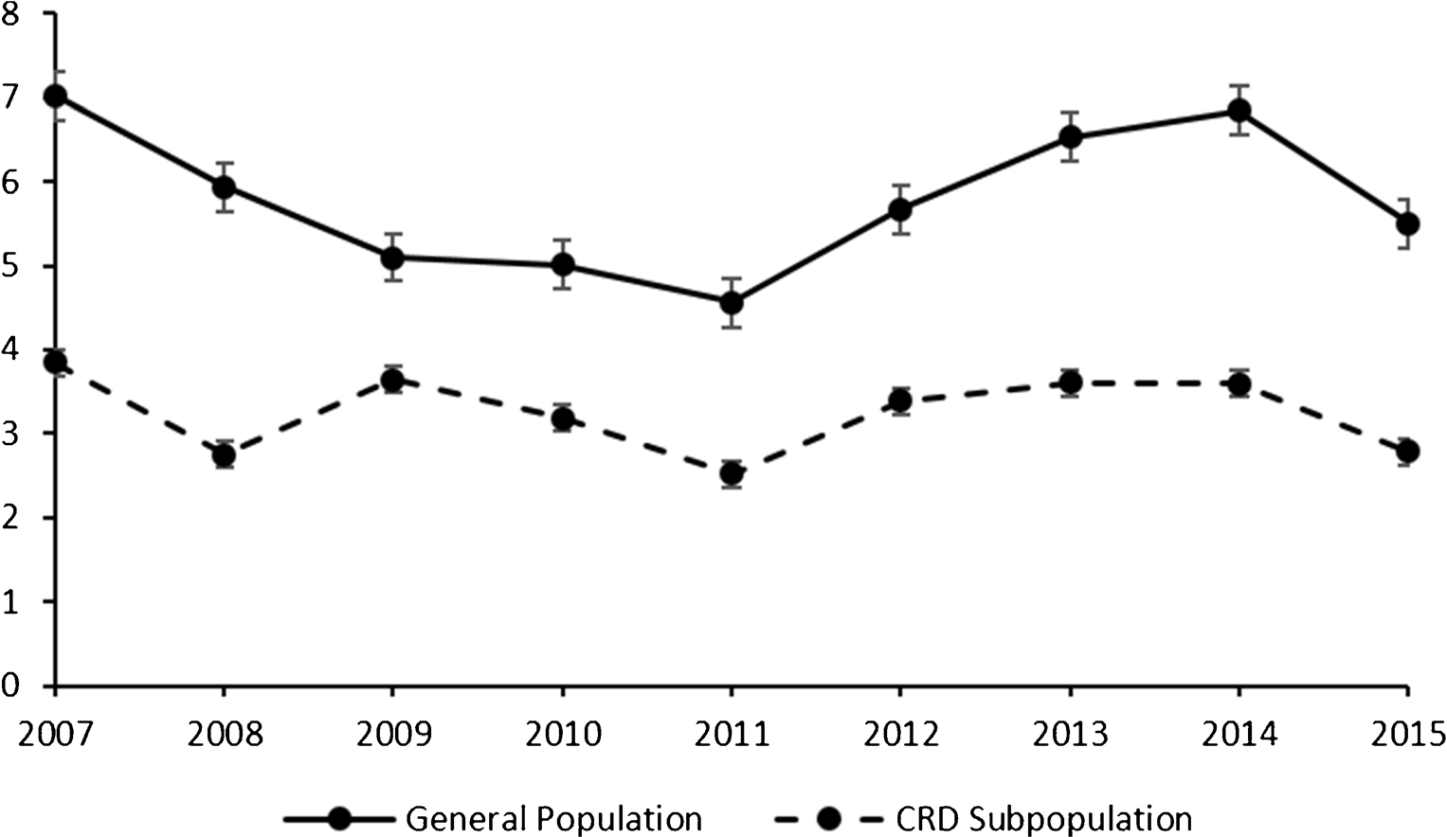
Annual incidence rate ratios (IRR) for all-cause secondary care utilisation comparing general population (solid) non-tuberculous mycobacterial disease (NTMD) cases to non-cases and chronic respiratory disease (CRD; dashed) subpopulation NTMD cases to non-cases. Secondary care utilisation comprising of inpatient, outpatient, and emergency visits. CRD including asthma, bronchiectasis, cystic fibrosis, chronic obstructive pulmonary disease, and interstitial lung disease. 95% confidence intervals shown

On average, secondary care utilisation was 5.80 (IRR; 95% CI 5.14, 6.46) higher for NTMD patients than non-NTMD patients, from 2007 to 2015 (Fig. 2).

### Secondary care utilisation in the CRD subpopulation

In the CRD subpopulation, average IRR from 2007 to 2015 comparing NTMD patients to non-NTMD patients was 3.26 (95% CI 2.89, 3.62) (Fig. 2).

## Discussion

This is the first study to assess NTMD risk factors and secondary care burden in the UK using a representative primary care adult population and a CRD subpopulation. Prior TB and bronchiectasis were most strongly associated with NTMD, while medications that suppressed the immune system were also strongly associated.

In this UK primary care population, prior TB was found to be the strongest risk factor; an association with previous TB has also been found in populations from Taiwan [23] and Denmark [3]. Patients with a history of TB may be more likely to have their sputum cultured for mycobacteria, increasing NTMD identification; however, it has also been proposed that post-inflammatory bronchiectasis resulting from TB may account for some increased risk [24].

Bronchiectasis was the most strongly associated CRD with NTMD, as seen elsewhere [3]. Previous research has explored the relationship between bronchiectasis and NTMD and it has been proposed that the two diseases are inextricably linked such that it may not be clear which disease preceded the other [7].

Asthma and ILD were not significantly associated with NTMD in our treatment-adjusted models. The odds of NTMD associated with COPD halved from the disease-only models to the treatment-adjusted models, suggesting the increased risk was in a large part due to COPD treatment. Patients with CRD may experience acute episodes of worsening symptoms, termed exacerbations, which may require additional medication and/or hospitalisation. Exacerbations are often provoked by infections, and treatment given (e.g. antibiotics, OCS) may be detrimental to those with NTM infection. Importantly, it has been shown that NTMD risk increases with increasing number of COPD exacerbations in the year prior to NTMD diagnosis [3]. Exacerbation treatment may similarly be driving risks seen due to asthma and ILD in studies not accounting for treatment [3, 11]. CRD patients considered to be at risk for NTMD may benefit from screening prior to being prescribed these therapies.

Many previous studies have pointed to immunosuppression as a risk factor for NTMD, including due to immunosuppressive medication [8] and steroids [4]. Immunosuppressive drugs, excluding corticosteroids, were significantly associated with NTMD, though more so in CRD patients. As in previous studies [3, 10], we found that ICS significantly increased odds of NTMD; however, OCS demonstrated an even greater association with NTMD. While ICS was significantly associated with NTMD, most probably by decreasing local immunity [3], immunosuppressive medications and OCS, which decrease systemic immunity, were more strongly associated with NTMD.

RA was significantly associated with NTMD in all models. Previous research has found that RA patients exposed to anti-TNF-α saw increased incidence of NTMD [25]. In our study, the odds of NTMD associated with RA halved from the disease-only to the treatment-adjusted model. Immunosuppressive therapy appears to play a significant role in the association of RA and NTMD; however, more studies are needed to determine if RA itself is a risk factor.

As seen previously, smoking was not significantly associated with NTMD [4] while being underweight was [4, 6]. Unlike previous studies [4, 8], we found diabetes increased risk of NTMD. Diabetes has been shown to be a risk factor for TB and likely through similar mechanisms may increase NTMD risk [26]. Previous studies found a higher proportion of CKD [10, 11] and GORD [11] in NTMD patients than in controls, but have not examined these as risk factors. CKD was significantly associated with NTMD, but was not significant in CRD patients; while GORD does not appear to be related to any increase in NTMD risk, as seen here and in a US study [8]. Structural damage in the lungs, combined with immunosuppression, may contribute to increased NTMD risk due to lung cancer, but not other cancers. A previous study found increased odds of NTMD with the presence of malignancy [27]; however, it did not break malignancy into types.

On average, following their diagnosis of NTMD, patients experienced nearly six times more all-cause secondary care events than patients without NTMD. This is higher than previous US and German studies, which found that patients with NTMD experience two to three times more hospitalisations, respectively, than general population controls without NTMD [12, 13]. Neither study investigated secondary care utilisation of NTMD patients with CRDs versus CRD patients without NTMD, meaning bias could have been introduced due to their cases having more CRD at baseline than their controls. In our study, those CRD patients with NTMD experienced over three times more all-cause secondary care events than those CRD patients without NTMD. Demonstrating that having NTMD increases secondary care events regardless of CRD status.

### Limitations

We did not have linked microbiological data therefore misclassification of NTMD was possible. We took a number of steps to ensure that those included were very likely to have NTMD, although it is possible some patients with disease were not included in our study. Firstly, we identified patients taking multi-drug combinations that are guideline-recommended for the treatment of NTMD, as well as patients on other combinations not explicitly recommended, but consisting of recommended drugs [5, 16, 17]. Secondly, we excluded all patients taking any multi-drug regimen for < 3 months. Thirdly, we excluded patients taking rifampicin in the absence of ethambutol or isoniazid; rifampicin can be used to treat other infections [28] and recommended regimens for NTMD have rifampicin paired with at least one other antimycobacterial [5, 16, 17]. Lastly, we excluded patients with < 3 sputum samples sent for NTM as three is the guideline-recommended number of samples to be taken, with at least two of these being positive for diagnosis of NTMD [5, 17]. It is possible that a small number of included patients were being treated with antimycobacterial drugs for other reasons; but, importantly, treatment for latent or active TB is unlikely in primary care [29]. Small numbers prevented analyses of NTMD mortality. As expected, small numbers prevented detailed analyses of CF in our exclusively adult, primary care populations. Cases and controls in the CRD subpopulation were not matched on CRD diagnoses, meaning the distribution of CRDs within cases and controls was different and may have led to bias.

## Conclusions

While prospective screening for NTMD is recommended for prior TB [17] and bronchiectasis [30], it is not explicitly recommended for other patients being considered for biologic therapies, including ICS and OCS used in other CRDs. These patients may potentially benefit from further clinical investigations prior to the start of biologic therapies, as these appear to substantially increase the risk of NTMD.
